# Establishment of EMab-134, a Sensitive and Specific Anti-Epidermal Growth Factor Receptor Monoclonal Antibody for Detecting Squamous Cell Carcinoma Cells of the Oral Cavity

**DOI:** 10.1089/mab.2017.0042

**Published:** 2017-12-01

**Authors:** Shunsuke Itai, Shinji Yamada, Mika K. Kaneko, Yao-Wen Chang, Hiroyuki Harada, Yukinari Kato

**Affiliations:** ^1^ Department of Antibody Drug Development, Tohoku University Graduate School of Medicine, Sendai, Japan.; ^2^ Department of Oral and Maxillofacial Surgery, Graduate School of Medical and Dental Sciences, Tokyo Medical and Dental University, Tokyo, Japan.; ^3^ New Industry Creation Hatchery Center, Tohoku University, Sendai, Japan.

**Keywords:** EGFR, monoclonal antibody, immunohistochemistry, oral cancer

## Abstract

Epidermal growth factor receptor (EGFR), a receptor tyrosine kinase, activates downstream signaling cascades in many tumors. In this study, we established novel anti-EGFR monoclonal antibodies (mAbs) and characterized their efficacy in flow cytometry, Western blot, and immunohistochemical analyses. We immunized mice with a combination of the extracellular domain of EGFR and EGFR-overexpressing LN229 glioblastoma cells (LN229/EGFR) and performed the first screening using enzyme-linked immunosorbent assay. Next, we selected mAbs using flow cytometry. Among 156 established clones, two mAbs, EMab-51 (IgG_1_, kappa) and EMab-134 (IgG_1_, kappa), reacted with EGFR in Western blot analysis; EMab-134 showed a much higher sensitivity compared with EMab-51. We compared the binding affinities of EMab-51 and EMab-134 using flow cytometry; the calculated *K*_D_ values for EMab-51 and EMab-134 against SAS cells/HSC-2 cells were 9.2 × 10^−9^ M/9.9 × 10^−9^ M and 2.6 × 10^−9^ M/8.3 × 10^−9^ M, respectively, indicating that EMab-134 has a higher affinity to EGFR-expressing cells. Immunohistochemical analysis of EMab-51 and EMab-134 showed sensitive and specific reactions against oral cancer cells; EMab-134 demonstrated a much higher sensitivity (36/38 cases; 94.7%) to oral squamous cell carcinomas compared with EMab-51 (6/38 cases; 15.8%). This novel anti-EGFR mAb, EMab-134, could be advantageous for detecting EGFR in the pathological analysis of EGFR-expressing cancers.

## Introduction

Epidermal growth factor receptor (EGFR) is a member of the human EGFR (HER) family of receptor tyrosine kinases.^(1–3)^ EGFR forms homodimers or heterodimers with other members of the HER family, such as HER2^(4)^ and HER3,^(5)^ controlling many biological processes. EGFR is a type-I transmembrane glycoprotein that is involved in cell growth and differentiation.^(6)^ Overexpression of EGFR is observed in many cancers, including head and neck, lung, colorectal, breast, pancreatic, kidney, ovary, bladder, and prostate cancers.^(7)^

Monoclonal antibodies (mAbs) have been developed for cancer treatment, including cetuximab (a mouse–human chimeric mAb; IgG_1_) against head and neck and colorectal cancers, panitumumab (a fully human mAb; IgG_2_) against colorectal cancers, and necitumumab (a fully human mAb; IgG_1_) against non-small cell lung cancers.^(8–10)^ Anti-EGFR mAbs possess various functional mechanisms: antibody-dependent cellular cytotoxicity (ADCC), complement-dependent cytotoxicity (CDC), blocking dimerization, blocking ligand binding, and EGFR endocytosis.

Recently, we developed anti-HER2 mAb (clone: H_2_Mab-77) using our original technology. H_2_Mab-77 is useful for Western blot, flow cytometry, and immunohistochemical analyses.^(11)^ In this study, we established sensitive and specific mAbs against EGFR.

## Materials and Methods

### Cell lines

Chinese hamster ovary (CHO)-K1, P3X63Ag8U.1 (P3U1), HEK-293T, Met-5A, LN229, and A431 were obtained from the American Type Culture Collection (ATCC; Manassas, VA). HSC-2, HSC-3, HSC-4, HSC-3M3, Ca9-22, HO-1-u-1, and SAS were obtained from the Japanese Collection of Research Bioresources Cell Bank (Osaka, Japan). LN229/EGFR and CHO/EGFR were produced by transfecting pCAG/PA-EGFR-RAP-MAP into LN229 and CHO-K1 cells using the Neon transfection system (Thermo Fisher Scientific, Inc., Waltham, MA) and Lipofectamine LTX (Thermo Fisher Scientific, Inc.), respectively.^(12)^ A few days after transfection, PA tag-positive cells were sorted using a cell sorter (SH800; Sony Corp., Tokyo, Japan). The PA tag system comprises a rat anti-human podoplanin mAb (clone NZ-1) and the PA tag (GVAMPGAEDDVV) derived from the platelet aggregation-stimulating (PLAG) domain of human podoplanin.^(13)^

### Animals and tissues

Four-week-old female BALB/c mice were purchased from CLEA Japan (Tokyo, Japan). Animals were housed under specific pathogen-free conditions. The Animal Care and Use Committee of Tohoku University approved all of the animal experiments described. Oral cancer tissue arrays were purchased from US Biomax, Inc. (Rockville, MD).

### Culture of cell lines

CHO-K1, CHO/EGFR, and P3U1 cell lines were cultured in RPMI 1640 medium (Nacalai Tesque, Inc., Kyoto, Japan), and LN229, LN229/EGFR, A431, HSC-2, HSC-3, HSC-4, HSC-3M3, Ca9-22, HO-1-u-1, SAS, HEK-293T, and Met-5A cell lines were cultured in Dulbecco's modified Eagle's medium (DMEM) (Nacalai Tesque, Inc.), supplemented with 10% heat-inactivated fetal bovine serum (Thermo Fisher Scientific, Inc.), 100 U/mL of penicillin, 100 μg/mL of streptomycin, and 25 μg/mL of amphotericin B (Nacalai Tesque, Inc.) at 37°C in a humidified atmosphere containing 5% CO_2_ and 95% air.

### Purification of extracellular domain of EGFR

The extracellular domain of EGFR with N-terminal PA tag and C-terminal RAP tag-MAP tag was purified from the supernatant of LN229/sol-EGFR using anti-RAP tag, as described previously.^(14)^ The RAP tag system comprises a mouse anti-rat podoplanin mAb (clone PMab-2) and the RAP tag (DMVNPGLEDRIE) derived from the PLAG domain of rat podoplanin.^(14)^

### Production of hybridoma cell lines

BALB/c mice were immunized using intraperitoneal injections of LN229/EGFR cells or 100 μg of sol-EGFR together with Imject Alum (Thermo Fisher Scientific, Inc.). After several additional immunizations, a booster injection of LN229/EGFR cells or 100 μg of sol-EGFR was intraperitoneally administered 2 days before harvesting spleen cells. Spleen cells were then fused with P3U1 cells using PEG1500 (Roche Diagnostics, Indianapolis, IN) or GenomONE-CF (Ishihara Sangyo Kaisha, Ltd., Osaka, Japan). The resulting hybridomas were grown in RPMI 1640 medium supplemented with hypoxanthine, aminopterin, and thymidine selection medium supplement (Thermo Fisher Scientific, Inc.). Culture supernatants were screened using enzyme-linked immunosorbent assay (ELISA) with sol-EGFR, and mAbs were selected using flow cytometry, Western blot, and immunohistochemical analyses.

### Purification of mAbs

Hybridomas were cultured in Hybridoma-SFM medium (Thermo Fisher Scientific, Inc.). mAbs were purified from supernatants using Protein G Sepharose 4 Fast Flow (GE Healthcare UK Ltd., Buckinghamshire, England).

### Flow cytometry

Cells were harvested by brief exposure to 0.25% trypsin/1-mM ethylenediaminetetraacetic acid (EDTA) (Nacalai Tesque, Inc.). After washing with 0.1% bovine serum albumin (BSA)/phosphate buffered saline (PBS), the cells were treated with 1 μg/mL of anti-EGFR for 30 minutes at 4°C, followed by treatment with Alexa Fluor 488-conjugated anti-mouse IgG (1:1000; Cell Signaling Technology, Inc., Danvers, MA). Fluorescence data were collected using EC800 Cell Analyzers (Sony Corp.).

### Determination of the binding affinity using flow cytometry

SAS and HSC-2 (2 × 10^5^ cells) were suspended in 100 μL of serially diluted mAbs (0.6 ng/mL–10 μg/mL), and Alexa Fluor 488-conjugated anti-mouse IgG (1:1000; Cell Signaling Technology, Inc.) was then added. Fluorescence data were collected using a cell analyzer (EC800; Sony Corp.). The dissociation constant (*K*_D_) was calculated by fitting the binding isotherms using the built-in one-site binding models in GraphPad PRISM 6 (GraphPad Software, Inc., La Jolla, CA).

### Western blot analysis

Cell lysates (10 μg) were boiled in SDS sample buffer (Nacalai Tesque, Inc.). Proteins were then electrophoresed on 5%–20% polyacrylamide gels (Wako Pure Chemical Industries Ltd., Osaka, Japan) and transferred onto polyvinylidene difluoride (PVDF) membranes (Merck KGaA, Darmstadt, Germany). After blocking with 4% skim milk (Nacalai Tesque, Inc.), membranes were incubated with anti-EGFR (1–10 μg/mL) and 1 μg/mL of anti-β-actin (clone AC-15; Sigma-Aldrich Corp., St. Louis, MO), followed by incubation with peroxidase-conjugated anti-mouse IgG (diluted 1:1000; Agilent Technologies, Inc., Santa Clara, CA), and were finally developed using ImmunoStar LD (Wako Pure Chemical Industries Ltd.) using a Sayaca-Imager (DRC Co. Ltd., Tokyo, Japan).

### Immunohistochemical analyses

Histological sections (4-μm thick) were directly autoclaved in EnVision FLEX Target Retrieval Solution High pH (Agilent Technologies, Inc.) for 20 minutes or were deparaffinized in xylene and then rehydrated and autoclaved in citrate buffer, pH 6.0 (Agilent Technologies, Inc.) for 20 minutes. Sections were then incubated with 5 μg/mL of mAbs for 1 hour at room temperature, followed by treatment with an Envision+ kit (Agilent Technologies, Inc.) for 30 minutes. Color was developed using 3,3-diaminobenzidine tetrahydrochloride (Agilent Technologies, Inc.) for 2 minutes, and sections were then counterstained with hematoxylin (Wako Pure Chemical Industries Ltd.). The intensity of staining was evaluated as 0, 1+, 2+, or 3+.

## Results

### Establishment of anti-EGFR mAbs

EGFR is a type-I transmembrane glycoprotein, which is similar to the platelet aggregation-inducing factor podoplanin.^(15)^ We previously produced anti-podoplanin cancer-specific mAbs (CasMabs), clones LpMab-2^(16,17)^ and LpMab-23,^(18,19)^ which particularly recognized cancer-type podoplanin. It is critical that immunogens are produced using cancer cell lines, such as LN229 glioblastoma cells, which express cancer-specific glycan-attached membrane proteins. We have used this method to develop useful mAbs against many membrane proteins.^(20)^

In this study, we immunized mice with LN229/EGFR or purified recombinant EGFR (sol-EGFR). Culture supernatants were then screened for binding to sol-EGFR using ELISA. We then used flow cytometry as a second screen to assess reactions with LN229 and LN229/EGFR cells. A stronger reaction against LN229/EGFR was required because LN229 cells express endogenous EGFR.^(21)^ Of 156 established clones, only two mAbs (1.3%), EMab-51 (IgG_1_, kappa) and EMab-134 (IgG_1_, kappa), reacted with EGFR in Western blot analysis. Both mAbs were produced by immunizing sol-EGFR. We purified EMab-51 and EMab-134 using Protein G and characterized these mAbs.

### Characterization of anti-EGFR mAbs

**FIG. 1.**
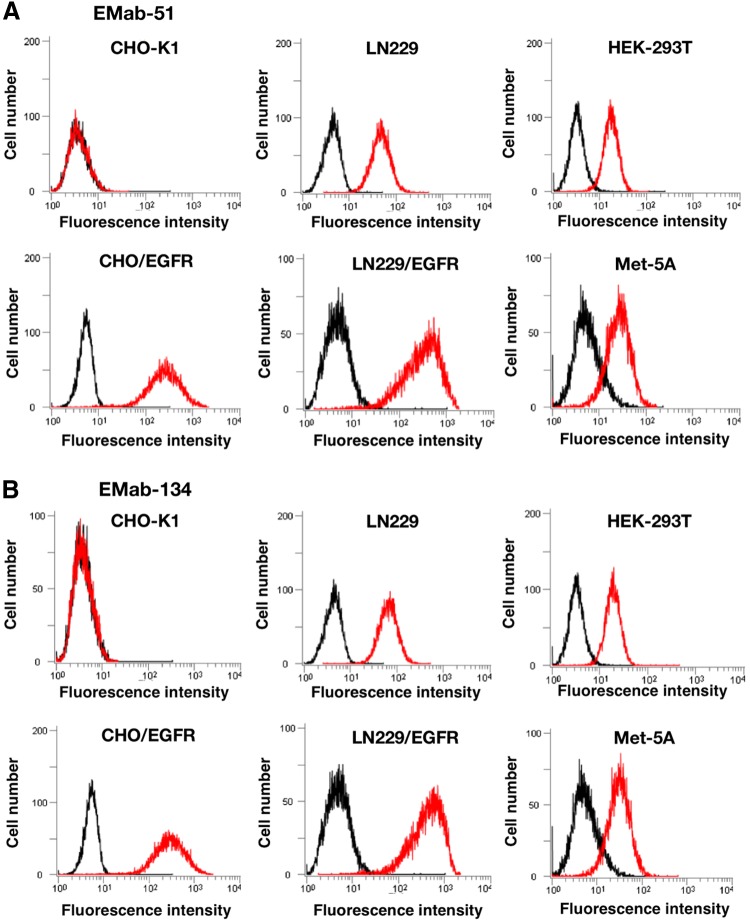
Flow cytometry using anti-EGFR mAbs for several cells. Cells were treated with 1 μg/mL of EMab-51 **(A)** and EMab-134 **(B)**, followed by treatment with Alexa Fluor 488-conjugated anti-mouse IgG; black line, negative control. EGFR, epidermal growth factor receptor; mAbs, monoclonal antibodies.

In flow cytometry, EMab-51 reacted with CHO/EGFR but not with the parental cell CHO-K1, indicating that EMab-51 had specificity for EGFR (Fig. 1A). EMab-51 also reacted with LN229/EGFR more strongly than with endogenous EGFR-expressing LN229 glioblastoma cells (Fig. 1A). Moreover, EMab-51 reacted with normal cell lines, including HEK-293T (renal epithelial cells) and Met-5A (mesothelial cells).^(22)^ The reactions of EMab-134 were very similar to those of EMab-51 (Fig. 1B).

**FIG. 2.**
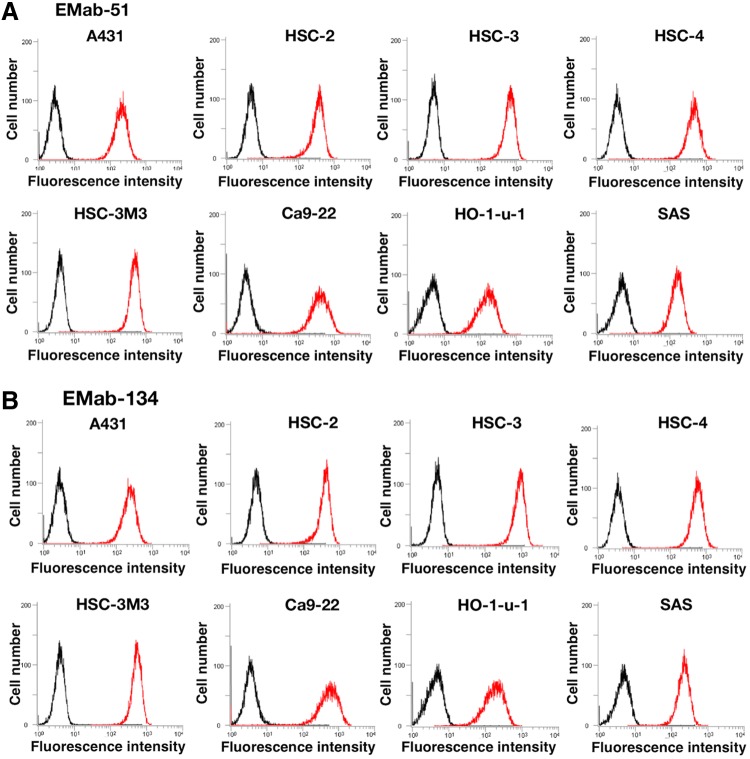
Flow cytometry using anti-EGFR mAbs for squamous cell carcinoma cell lines. Cells were treated with 1 μg/mL of EMab-51 **(A)** and EMab-134 **(B)**, followed by treatment with Alexa Fluor 488-conjugated anti-mouse IgG; black line, negative control.

EMab-51 recognized endogenous EGFR in A431 epidermoid carcinoma cells^(23)^ (Fig. 2A). EMab-51 also reacted with oral squamous cell carcinomas such as HSC-2, HSC-3, HSC-4, HSC-3M3, Ca9-22, HO-1-u-1, and SAS, indicating that EMab-51 detected each squamous cell carcinoma cell line at a low concentration (1 μg/mL) in flow cytometry. Another clone, EMab-134, also reacted with squamous cell carcinoma cell lines in the same way as EMab-51 (Fig. 2B).

**FIG. 3.**
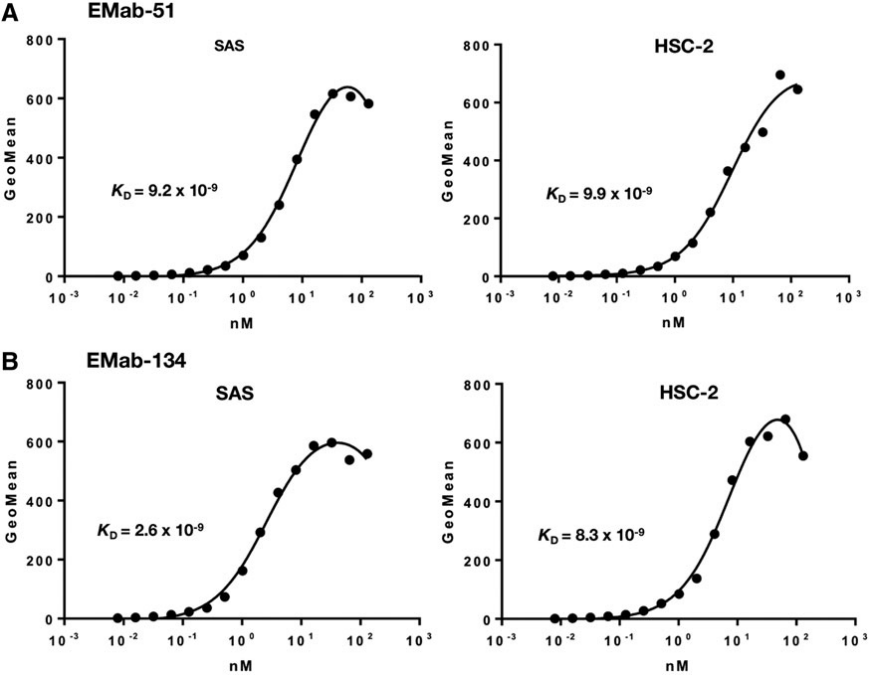
Determination of binding affinity using flow cytometry. SAS and HSC-2 were suspended in 100 μL of serially diluted EMab-51 **(A)** or EMab-134 **(B)** (0.6 ng/mL–10 μg/mL), and secondary anti-mouse IgG was then added. Fluorescence data were collected using a cell analyzer.

We further determined the binding affinities of EMab-51 and EMab-134 for SAS and HSC-2 cells through flow cytometry (Fig. 3) and calculated *K*_D_ values for EMab-51 and EMab-134. *K*_D_ values of EMab-51 were determined to be 9.2 × 10^−9^ M and 9.9 × 10^−9^ M against SAS and HSC-2, respectively. In contrast, *K*_D_ values of EMab-134 were determined to be 2.6 × 10^−9^ M and 8.3 × 10^−9^ M against SAS and HSC-2, respectively, indicating that the binding affinities of EMab-134 were 3.5 times and 1.2 times higher than those of EMab-51 against SAS and HSC-2, respectively.

**FIG. 4.**
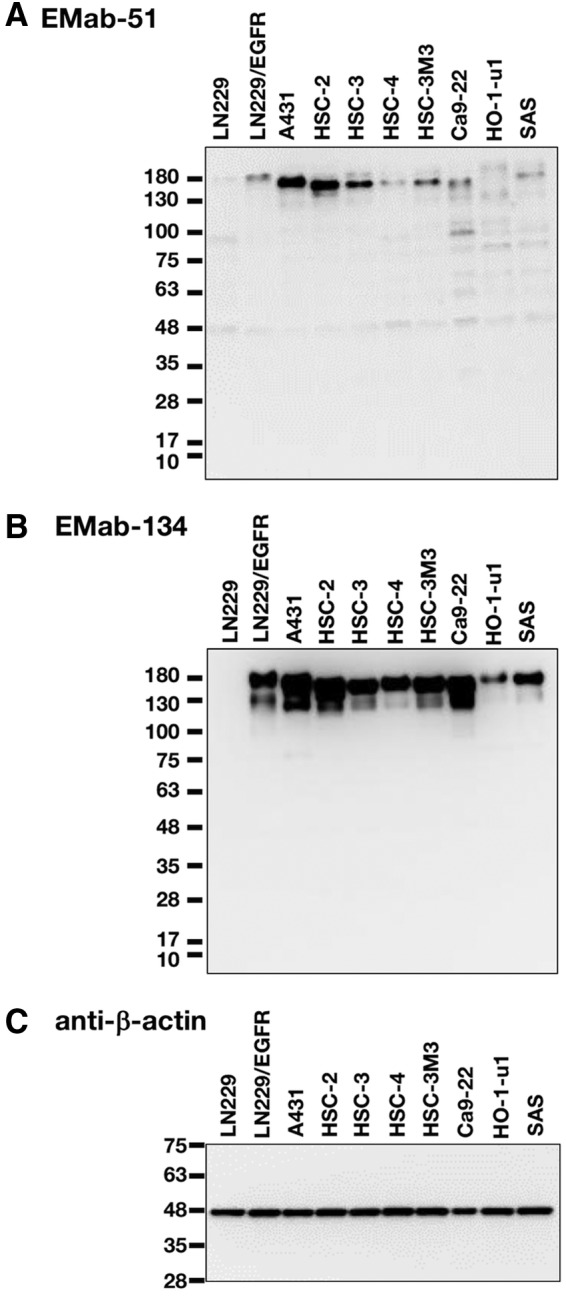
Western blot analysis using EMab-51 and EMab-134. Cell lysates (10 μg) were electrophoresed, and proteins were transferred onto PVDF membranes. After blocking, membranes were incubated with 10 μg/mL of EMab-51 **(A)**, 1 μg/mL of EMab-134 **(B)**, or 1 μg/mL of anti-β-actin (clone: AC-15) **(C)** and then incubated with peroxidase-conjugated anti-mouse IgG. PVDF, polyvinylidene difluoride.

Next, we compared the reactivity between EMab-51 and EMab-134 in Western blot analysis. As shown in Figure 4, both EMab-51 and EMab-134 reacted with LN229/EGFR. However, the sensitivity of EMab-134 was much higher compared with EMab-51, although their reactivity was similar in flow cytometry (Figs. 1 and 2). Both mAbs reacted with endogenous EGFR, which was reported to be expressed in A431 cells.^(23)^ EMab-51 detected an ∼180-kDa weak signal in LN229/EGFR and a strong signal in A431 cells (Fig. 4A), whereas EMab-134 detected ∼180- and 130-kDa signals in LN229/EGFR and A431 cells, respectively (Fig. 4B). Furthermore, EMab-134 showed strong signals against five human oral cancer cell lines (HSC-2, HSC-3, HSC-4, HSC-3M3, and Ca9-22) and moderate signals against two human oral cancer cell lines (HO-1-u-1 and SAS; Fig. 4B), which were compatible with the flow cytometric data (Fig. 3). In contrast, EMab-51 showed moderate signals against two cell lines (HSC-2 and HSC-3) and weak signals against the other cell lines (Fig. 4A).

### Immunohistochemical analysis for oral cancers

**FIG. 5.**
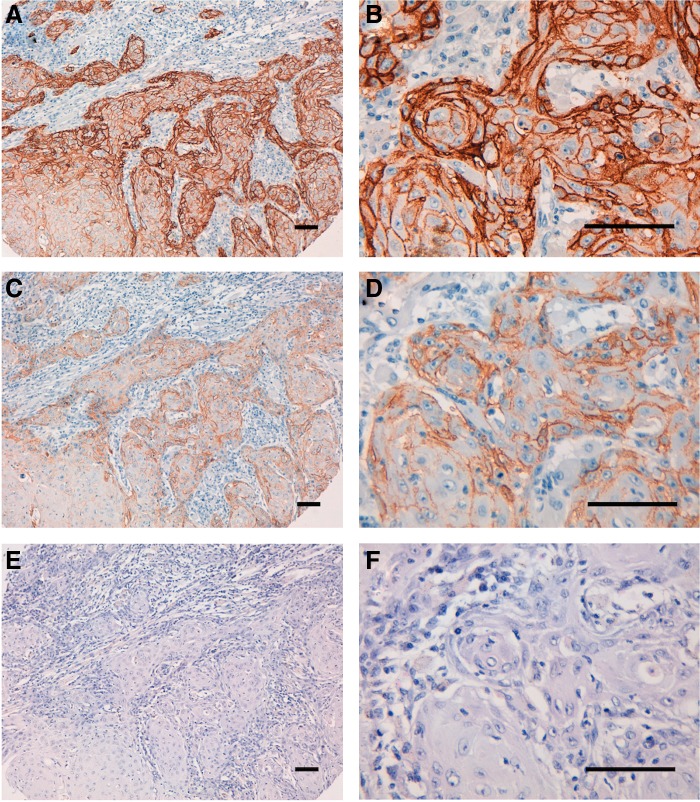
Immunohistochemical analysis by EMab-134 for oral cancers. After antigen retrieval, sections (Case-24) were incubated with 5 μg/mL of primary EMab-134 for 1 hour at room temperature, followed by treatment with Envision+ kit for 30 minutes. Color was developed using 3,3-diaminobenzidine tetrahydrochloride for 2 minutes, and sections were then counterstained with hematoxylin. **(A, B)** Antigen retrieval using EnVision FLEX Target Retrieval Solution High pH; **(C, D)** antigen retrieval using citrate buffer, pH 6.0; **(E, F)** hematoxylin and eosin staining; scale bar = 100 μm.

**FIG. 6.**
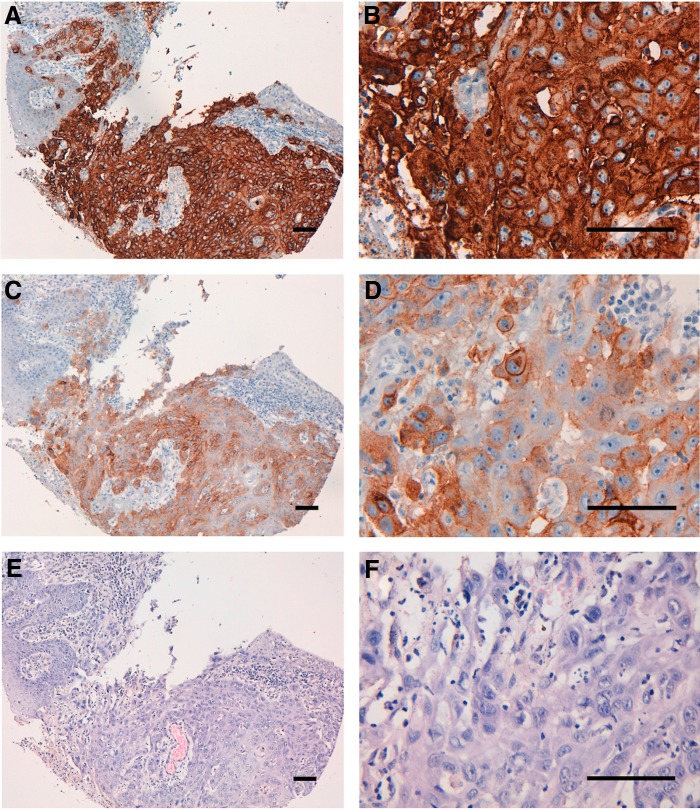
Immunohistochemical analysis by anti-EGFR mAbs for oral cancers. After antigen retrieval using EnVision FLEX Target Retrieval Solution High pH, sections (Case-15) were incubated with 5 μg/mL of primary EMab-134 or EMab-51 for 1 hour at room temperature, followed by treatment with Envision+ kit for 30 minutes. Color was developed using 3,3-diaminobenzidine tetrahydrochloride for 2 minutes, and sections were then counterstained with hematoxylin. **(A, B)** EMab-134; **(C, D)** EMab-51; **(E, F)** hematoxylin and eosin staining; scale bar = 100 μm.

We performed immunohistochemical analysis using EMab-134 for human oral cancers because EGFR expression was highly observed in oral cancer cell lines (Fig. 2). First, we investigated the antigen retrieval conditions for immunohistochemical analysis using EMab-134. EMab-134 stained the membranes of cancer cells of oral squamous cell carcinomas (Fig. 5A–D). The sensitivity using EnVision FLEX Target Retrieval Solution High pH (Fig. 5A, B and Supplementary Fig. S1A, B) was much higher than that using citrate buffer (pH 6.0) as the antigen retrieval solution (Fig. 5C, D and Supplementary Fig. S1C, D). Next, we compared the immunoreactivity of EMab-51 and EMab-134 under the same conditions (Fig. 6). EMab-134 (Fig. 6A, B) showed much stronger signals than EMab-51 (Fig. 6C, D), although the staining areas of EMab-134 and EMab-51 were the same. All staining results of EMab-51 and EMab-134 for oral cancers are shown in Table 1. EMab-51 stained 6/38 (15.8%) of squamous cell carcinomas, whereas EMab-134 stained 36/38 (94.7%) of the carcinomas (Table 2). Several typical results of EMab-134 are also shown in Supplementary Figures S2 (intensity: 3+) and S3 (intensity: 2+).

**Table 1.** Results of EMab-51 and EMab-134 Immunostaining in 48 Patients with Oral Cancers

*No.*	*Sex*	*Age*	*Organ*	*Pathology diagnosis*	*Differentiation*	*Type*	*EMab-51*	*EMab-134*
1	M	61	Tongue	SCC	Well	Malignant	0	0
2	F	57	Tongue	SCC	Well	Malignant	0	1+
3	F	67	Tongue	SCC	Well	Malignant	0	2+
4	M	59	Tongue	SCC	Well	Malignant	0	1+
5	F	47	Tongue	SCC	Well	Malignant	0	1+
6	F	62	Tongue	SCC	—	Malignant	0	3+
7	F	47	Tongue	SCC	Well	Malignant	0	2+
8	M	51	Tongue	SCC	Well	Malignant	0	0
9	M	62	Tongue	SCC	Well	Malignant	0	2+
10	F	53	Tongue	SCC	Well	Malignant	0	3+
11	M	50	Tongue	SCC	Well	Malignant	0	3+
12	M	76	Tongue	SCC	Well	Malignant	0	3+
13	M	55	Tongue	SCC	Well	Malignant	0	1+
14	F	57	Tongue	SCC	Moderately	Malignant	0	1+
15	M	61	Tongue	SCC	Well	Malignant	3+	3+
16	F	50	Tongue	SCC	Well	Malignant	0	2+
17	M	54	Tongue	SCC	Well	Malignant	0	2+
18	M	62	Tongue	SCC	Well	Malignant	0	1+
19	F	55	Tongue	SCC	Well	Malignant	2+	3+
20	F	63	Tongue	SCC	Well	Malignant	0	1+
21	M	56	Tongue	SCC	Well	Malignant	0	1+
22	F	45	Tongue	SCC	Well	Malignant	0	3+
23	M	50	Tongue	SCC	Well	Malignant	2+	3+
24	F	46	Tongue	SCC	Poorly	Malignant	0	3+
25	F	48	Tongue	SCC	Moderately	Malignant	0	2+
26	F	67	Tongue	SCC	—	Malignant	0	2+
27	M	64	Tongue	SCC	Poorly	Malignant	1+	3+
28	F	50	Tongue	SCC	Moderately	Malignant	0	1+
29	M	63	Tongue	SCC	Moderately	Malignant	0	2+
30	F	46	Tongue	SCC	Well	Malignant	0	2+
31	F	35	Tongue	SCC	Moderately	Malignant	0	2+
32	M	55	Tongue	SCC	Poorly	Malignant	0	1+
33	M	49	Tongue	SCC	Poorly	Malignant	0	2+
34	M	61	Tongue	SCC	Moderately	Malignant	0	1+
35	M	53	Tongue	SCC	Moderately	Malignant	1+	3+
36	F	51	Tongue	SCC	Poorly	Malignant	0	2+
37	M	73	Tongue	SCC	Poorly	Malignant	0	2+
38	M	61	Tongue	SCC	Poorly	Malignant	1+	3+
39	M	62	Tongue	ACC	—	Malignant	0	0
40	M	50	Tongue	MC	—	Malignant	0	0
41	M	68	Parotid	PA	—	Malignant	0	1+
42	M	58	Parotid	PA	—	Malignant	0	0
43	M	41	Parotid	PA	—	Malignant	0	0
44	F	45	Parotid	MPA	—	Malignant	0	0
45	M	57	Parotid	MPA	—	Malignant	0	1+
46	M	52	Parotid	MPA	—	Malignant	0	1+
47	M	65	Parotid	AL	—	Malignant	0	1+
48	F	74	Parotid	ACC	—	Malignant	0	1+

ACC, adenoid cystic carcinoma; AL, adenolymphoma; F, female; M, male; MC, mucoepidermoid carcinoma; MPA, malignant pleomorphic adenoma; PA, pleomorphic adenoma; SCC, squamous cell carcinoma.

**Table 2.** Summary of Immunostaining Using EMab-51 and EMab-134

		*EMab-51 immunostaining*					*EMab-134 immunostaining*				
*Tumor type*	*No. of cases*	*3+*	*2+*	*1+*	*0*	*No. of positive cases (%)*	*3+*	*2+*	*1+*	*0*	*No. of positive cases (%)*
SCC	38	1	2	3	32	6/38 (15.8)	12	13	11	2	36/38 (94.7)
PA	3	0	0	0	3	0/3 (0)	0	0	1	2	1/3 (33.3)
MPA	3	0	0	0	3	0/3 (0)	0	0	2	1	2/3 (66.7)
ACC	2	0	0	0	2	0/2 (0)	0	0	1	1	1/2 (50)
MC	1	0	0	0	1	0/1 (0)	0	0	0	1	0/1 (0)
AL	1	0	0	0	1	0/1 (0)	0	0	1	0	1/1 (100)

## Discussion

To date, we have developed many mAbs against membrane proteins. We recently developed specific and sensitive anti-podocalyxin mAbs using a technology developed by us and demonstrated that the clone PcMab-47 was the most useful for flow cytometry, Western blot, and immunohistochemical analyses among the 100 anti-podocalyxin mAb clones. PcMab-47 has been useful for investigations of podocalyxin expression and function in cancers and normal tissues.^(20)^ We also used our technology to generate mAbs that bind to various novel epitopes of podoplanin, including LpMab-3,^(24)^ LpMab-12,^(25)^ LpMab-19,^(26)^ and LpMab-21.^(27,28)^ Importantly, these mAbs are useful for Western blot, flow cytometry, and immunohistochemical analyses for podoplanin. Recently, we further developed anti-HER2 mAb (clone: H_2_Mab-77), which is also useful for Western blot, flow cytometry, and immunohistochemical analyses.^(11)^ Development of mAbs, which are useful for various experiments, is often difficult because the design of immunogens and screening methods to produce specific mAbs differ based on the applications. Furthermore, it is difficult to produce mAbs sensitive and specific to endogenous proteins.

In this study, we immunized mice with a combination of LN229/EGFR and recombinant EGFR to develop specific and sensitive anti-EGFR mAbs, which were useful for Western blotting, flow cytometry, and immunohistochemical analyses. Two established clones, EMab-51 and EMab-134, were determined to be of the IgG_1_ subclass, precluding confirmation of ADCC or CDC without conversion to mouse IgG_2a_, mouse IgG_2b_, or human IgG_1_. In future studies, we will convert the subclass of EMab-51 and EMab-134 into ADCC/CDC-inducing subclasses for measuring ADCC/CDC activities.^(17,29,30)^ Furthermore, we should determine the epitope of EMab-134 and EMab-51 and investigate the reason why EMab-134 is sensitive in Western blot and immunohistochemical analyses.

In conclusion, of 156 clones of anti-EGFR mAbs, EMab-134 was highly efficacious in Western blot analysis and strongly stained oral cancers. Thus, EMab-134 could be useful in various experiments and advantageous for the pathological identification of EGFR in many cancers.

## Supplementary Material

Supplemental data

Supplemental data

Supplemental data
