# Supplementary material for: Establishment of EMab-134, a Sensitive and Specific Anti-Epidermal Growth Factor Receptor Monoclonal Antibody for Detecting Squamous Cell Carcinoma Cells of the Oral Cavity
Source: Monoclon Antib Immunodiagn Immunother. 2017 Dec 1;36(6):272–81. doi: 10.1089/mab.2017.0042 (PMC6975130; doi:10.1089/mab.2017.0042)
Supplement: Supplemental data [file Suppl_Fig1.pdf]

Supplementary Data

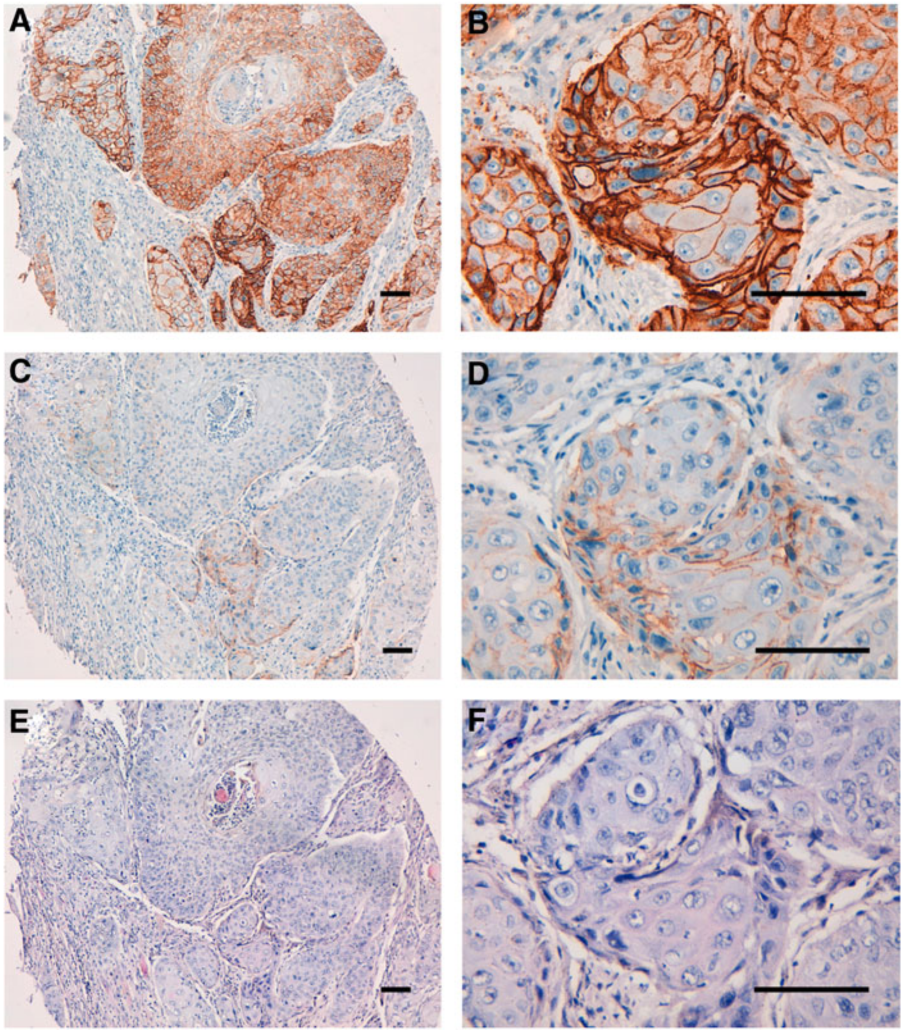

SUPPLEMENTARY FIG. S1. Immunohistochemical analysis by EMab-134 for oral cancers. After antigen retrieval, sections (Case 11) were incubated with 5 μg/mL of primary EMab-134 for 1 hour at room temperature, followed by treatment with Envision+ kit for 30 minutes. Color was developed using 3,3-diaminobenzidine tetrahydrochloride for 2 minutes, and sections were then counterstained with hematoxylin. **(A, B)** Antigen retrieval using EnVision FLEX Target Retrieval Solution High pH; **(C, D)** antigen retrieval using citrate buffer, pH 6.0; **(E, F)** hematoxylin and eosin staining; scale bar = 100 μm.
