# Supplementary material for: Establishment of EMab-134, a Sensitive and Specific Anti-Epidermal Growth Factor Receptor Monoclonal Antibody for Detecting Squamous Cell Carcinoma Cells of the Oral Cavity
Source: Monoclon Antib Immunodiagn Immunother. 2017 Dec 1;36(6):272–81. doi: 10.1089/mab.2017.0042 (PMC6975130; doi:10.1089/mab.2017.0042)
Supplement: Supplemental data [file Suppl_Fig3.pdf]

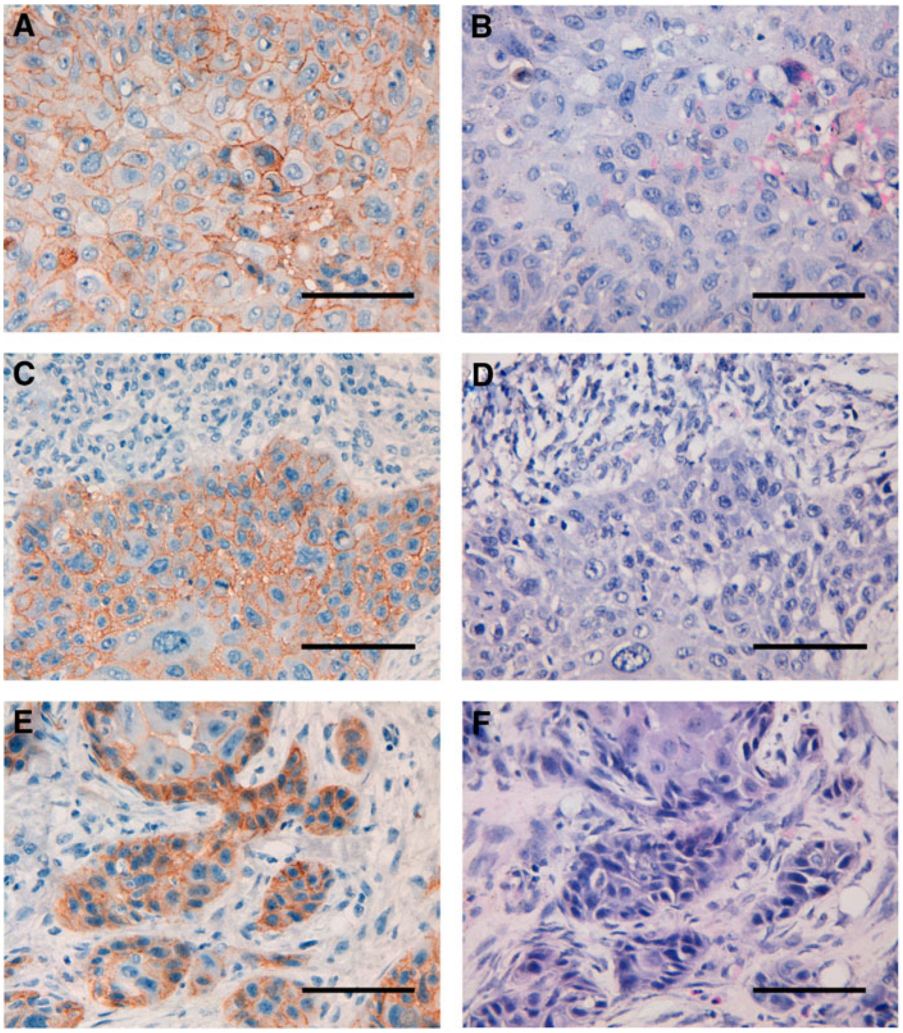

SUPPLEMENTARY FIG. S3. Immunohistochemical analysis by EMab-134 for oral cancers. **(A, C, E)** After antigen retrieval using EnVision FLEX Target Retrieval Solution High pH, sections were incubated with 5 μg/mL of primary EMab-134 for 1 hour at room temperature followed by treatment with Envision+ kit for 30 minutes. Color was developed using 3,3-diaminobenzidine tetrahydrochloride for 2 minutes, and sections were then counterstained with hematoxylin. **(B, D, F)** Hematoxylin and eosin staining; scale bar = 100 μm. **(A, B)** Case 3, **(C, D)** Case 9, **(E, F)** Case 26.
